# Comparative genomic analysis of the human genome and six bat genomes using unsupervised machine learning: Mb-level CpG and TFBS islands

**DOI:** 10.1186/s12864-022-08664-9

**Published:** 2022-07-08

**Authors:** Yuki Iwasaki, Toshimichi Ikemura, Kennosuke Wada, Yoshiko Wada, Takashi Abe

**Affiliations:** 1 Department of Bioscience, Nagahama Institute of Bio-Science and Technology, Tamura-cho 1266, Nagahama-shi, Shiga-ken 526-0829 Japan; 2 Smart Information Systems, Faculty of Engineering, Niigata University, Niigata-ken, 950-2181 Japan

**Keywords:** Artificial intelligence, Machine learning, Oligonucleotide composition, Self-organizing map, Centromeric heterochromatin, SARS-CoV-2

## Abstract

**Background:**

Emerging infectious disease-causing RNA viruses, such as the SARS-CoV-2 and Ebola viruses, are thought to rely on bats as natural reservoir hosts. Since these zoonotic viruses pose a great threat to humans, it is important to characterize the bat genome from multiple perspectives. Unsupervised machine learning methods for extracting novel information from big sequence data without prior knowledge or particular models are highly desirable for obtaining unexpected insights. We previously established a batch-learning self-organizing map (BLSOM) of the oligonucleotide composition that reveals novel genome characteristics from big sequence data.

**Results:**

In this study, using the oligonucleotide BLSOM, we conducted a comparative genomic study of humans and six bat species. BLSOM is an explainable-type machine learning algorithm that reveals the diagnostic oligonucleotides contributing to sequence clustering (self-organization). When unsupervised machine learning reveals unexpected and/or characteristic features, these features can be studied in more detail via the much simpler and more direct standard distribution map method. Based on this combined strategy, we identified the Mb-level enrichment of CG dinucleotide (Mb-level CpG islands) around the termini of bat long-scaffold sequences. In addition, a class of CG-containing oligonucleotides were enriched in the centromeric and pericentromeric regions of human chromosomes. Oligonucleotides longer than tetranucleotides often represent binding motifs for a wide variety of proteins (e.g., transcription factor binding sequences (TFBSs)). By analyzing the penta- and hexanucleotide composition, we observed the evident enrichment of a wide range of hexanucleotide TFBSs in centromeric and pericentromeric heterochromatin regions on all human chromosomes.

**Conclusion:**

Function of transcription factors (TFs) beyond their known regulation of gene expression (e.g., TF-mediated looping interactions between two different genomic regions) has received wide attention. The Mb-level TFBS and CpG islands are thought to be involved in the large-scale nuclear organization, such as centromere and telomere clustering. TFBSs, which are enriched in centromeric and pericentromeric heterochromatin regions, are thought to play an important role in the formation of nuclear 3D structures. Our machine learning-based analysis will help us to understand the differential features of nuclear 3D structures in the human and bat genomes.

**Supplementary Information:**

The online version contains supplementary material available at 10.1186/s12864-022-08664-9.

## Background

Emerging infectious disease-causing RNA viruses, such as SARS-CoV-2 and MERS coronaviruses and Ebola viruses, are thought to rely on bats as natural reservoir hosts [1]. Several research groups, including ours, have shown that the mono- and short oligonucleotide composition of these virus genomes changes in a directional time-dependent manner after invading the human population [2–8]. One of the primary causes of these directional changes is thought to be higher levels of APOBEC enzyme activity in human hosts than in bats [9–11]. Given that bats are natural reservoir hosts of diverse emerging infectious disease viruses that pose a significant threat to humans, the accumulation of multifaceted knowledge about the differences between the bat and human genomes should be important for combating these harmful zoonotic viruses. Due to societal concerns and biological interests regarding bats, reference-quality genome sequences have been reported for six species [10], and these sequences were used in the present study.

The composition of short oligonucleotides has been called the “genome signature” [12]; in microorganisms, it differs among species, even species with the same genome G + C%. In higher vertebrates such as mammals, however, there are clear intragenomic differences in both the mononucleotide composition (e.g., isochores) [13] and the oligonucleotide compositions [14–16]. Here, we conducted comparative genomic analyses of the human genome and six bat genomes by focusing on the oligonucleotide composition. Notably, oligonucleotides sequences, particularly those longer than tetra- or pentanucleotide sequences, often correspond to binding motifs for a wide variety of proteins (e.g., transcription factor binding sequences (TFBSs)), and a large amount of accumulated knowledge about oligonucleotide functions is available for the human genome. Since oligonucleotide sequences with important biological functions (e.g., TFBSs) tend to be conserved during evolution, we will obtain novel knowledge about the bat genome by utilizing the accumulated information about oligonucleotide composition of the human genome [14–16]. As will be explained in more detail later, we have identified Mb-level CpG and TFBS islands in centromeric and pericentromeric regions in the human genome, and based on these findings combined with the analysis of Hi-C data for Mb-level interchromosomal interactions reported by Lieberman-Aiden E, et al. [17], we hypothesized that Mb-level islands play a role in centromere clustering in interphase nuclei and thus formation of large-scale nuclear 3D structures of genome DNA [16].

Machine learning is one of artificial intelligence (AI) technologies, and unsupervised machine learning that can extract unexpected insights from big data without prior knowledge or specific models are highly desirable for use in current genome research, as reviewed by Libbrecht and Noble [18] and Yang et al. [19]. We previously developed a batch-learning self-organizing map (BLSOM) of the oligonucleotide compositions that enables the separation (self-organization) of genomic fragments (e.g., 100 kb) by species and phylogeny [20–22]. Importantly, BLSOM is an explainable-type machine learning algorithm that can identify the diagnostic oligonucleotides that contribute to the classification (self-organization) of genomic sequences. The basic strategy applied in the present study is to let machine learning carry out the majority of initial knowledge discovery without presetting particular models or hypotheses. Then, by focusing on the unexpected and/or characteristic results obtained by machine learning, we successively examine its findings via more direct methods.

## Materials and methods

### BLSOM

The Kohonen self-organizing map (SOM), an unsupervised neural network algorithm, is a powerful tool for clustering and visualizing high-dimensional complex data in a two-dimensional map [23]. We modified the conventional SOM for genome informatics based on batch learning so that the learning process and the resulting map were independent of the order of data input [24]. The initial weight vectors were defined based on principal component analysis (PCA) rather than by using random values. The weight vectors (wij) were arranged in a two-dimensional lattice denoted by i (= 0, 1,…, I-1) and j (= 0, 1,…, J-1) and were set and updated as described previously [20, 24]; weights in the first dimension (I) were arranged into lattices corresponding to a width of five times the standard deviation (SD) (5 × σ1) of the first principal component, and the second dimension (J) was defined by the nearest integer greater than σ2/σ1 × I. Here, σ1 and σ2 were the standard deviations of the first and second principal components, respectively. Therefore, the ratio of the two axes of the obtained BLSOMs differs depending on the data being analyzed. A BLSOM program suitable for PC cluster systems is available on our website (http://bioinfo.ie.niigata-u.ac.jp/?BLSOM).

### Genome sequences and TFBS data

**Fig. 1:**
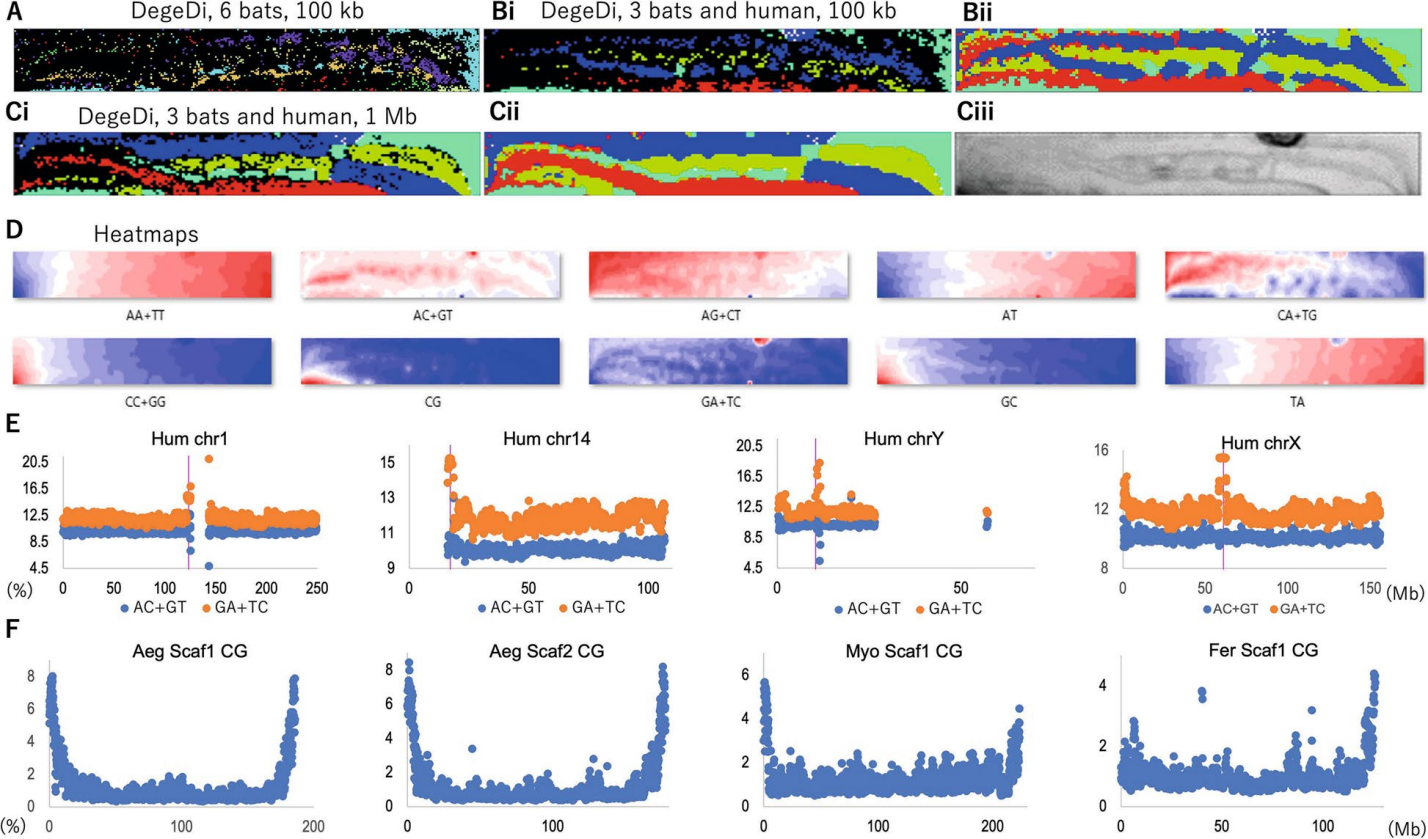
DegeDi-BLSOMs of bat and human genomes and the distribution of dinucleotides on their chromosomes. **A** BLSOM constructed for the DegeDi composition of 100-kb sequences in six bats. Nodes containing sequences from more than one species are indicated in black, and those containing sequences from a single species are indicated in color specific to the species: *Rousettus aegyptiacus* (Aeg) (  ), *Phyllostomus discolor* (Dis) (  ), *Rhinolophus ferrumequinum* (Fer) (  ), *Pipistrellus kuhlii* (Kuh) (  )*, Molossus molossus* (Mol) (  ) and *Myotis myotis* (Myo) (  ). The total number of nodes (grid points) was set to 1/20 of the total number of 100-kb sequences. **Bi** The BLSOM for the 100-kb DegeDi of three bats and humans was constructed and displayed as described in **A**. The colors representing the species are as follows: Aeg (  ), Fer (  ), Myo (  ) and Hum (  ). **Bii** Each grid point was colored according to the species with the highest value. **Ci** DegeDi-BLSOM for 1-Mb sequences with a 100-kb sliding step for three bats and humans. Each grid point is displayed as described in Bi. **Cii** Each grid point is displayed as described in Bii. **Ciii** U-matrix. **D** Heatmaps for the DegeDi-BLSOM described in **Ci**. **E** The occurrence frequency (%) of AC + GT and GA + TC in 1-Mb sliding windows with a 100-kb step is plotted on four human chromosomes. The central position of the primary constriction of each chromosome, as inferred from the UCSC Genome Brower (https://genome.ucsc.edu/), is indicated by the vertical magenta bar. **F** The occurrence frequency (%) of CG is plotted on four bat scaffolds

The sequences of six bat genomes published by Jebb et al. (2020) [10] were obtained from https://bds.mpi-cbg.de/hillerlab/Bat1KPilotProject/, on 2021/02/02; bat species names and their abbreviations are given in Fig. 1A legend. The *Homo sapiens* genome sequence (GRCh38/hg38) was obtained from the UCSC ftp site (http://hgdownload.soe.ucsc.edu/goldenPath/hg38/chromosomes/) on 2021/05/15. When the number of undetermined nucleotides (Ns) in a 100-kb or 1-Mb fragment sequence exceeded 20% of the fragment size, the sequence was omitted from the analysis. When the number of Ns was less than 20%, the oligonucleotide frequencies were normalized to the length without Ns and included in the analysis. Human TFBS sequences were obtained from the SwissRegulon Portal (http://swissregulon.unibas.ch/sr/downloads), which publishes TFBSs predicted by MotEvo: a computational tool that integrates Bayesian probabilistic methods for inferring regulatory sites and motifs based on multiple alignments of DNA sequences [25]. Since complementary oligonucleotides are treated as the same oligonucleotide in this study, we used 181 degenerate sets of hexanucleotide TFBSs (DegeHexa TFBSs) obtained from the SwissRegulon Portal (for the TFBS sequences, see Supplemental Table S1).

We confirmed that all methods were performed in accordance with the relevant guidelines and regulations.

## Results and discussion

### DegeDinucleotide BLSOM for bat and human genomes

The genome sequences registered in public DNA databases (e.g., International Nucleotide Sequence Database Collaboration; https://www.insdc.org/about) represent only one strand of complementary sequences, and the strand is chosen rather arbitrarily in the registration of fragment sequences, including scaffold sequences. In other words, the distinction between complementary sequences is usually not important for understanding the global characteristics of genome sequences. Here, two complementary nucleotides (e.g., AA and TT) are added together and referred to as degenerate dinucleotides (DegeDi) [21, 22]. Figure 1A shows a BLSOM of the DegeDi composition of 100-kb fragments from six bat genomes; the number of nodes is set so that an average of 20 sequences are attributed to each node (grid point). Importantly, no information other than the oligonucleotide composition is provided during machine learning (unsupervised learning). In the figure, nodes containing sequences of a single species are indicated by in the same color representing the species, and nodes containing sequences of multiple species are displayed in black. There are many black nodes, and the separation of each species is not good under these conditions, showing that the simultaneous use of six bat genomes complicates the results. Therefore, in constructing DegeDi-BLSOM for comparative genomic analyses including the human genome, we selected the following three bat species: the megabat *Rousettus aegyptiacus* (Aeg), the microbat *Rhinolophus ferrumequinum* (Fer) (which is closely related to the host species of the bat SARS strain that is thought to be the origin of SARS-CoV-2), and the microbat *Myotis myotis* (Myo). It should be noted here that sequences of the bats that are not used in the BLSOM analysis are used in the later distribution analysis of oligonucleotide frequencies.

Figure 1Bi shows the BLSOM results for the three bats and humans; in Fig. 1Bii, the sequences attributed to each node are colored according to the species with the largest number of sequences. The separation between bats and humans is not good under these analysis conditions, as shown in Fig. 1Bi. When comparing genomes, the short fragmentation of each genome is likely to complicate the BLSOM analysis for various reasons (some fragments primarily contain protein-coding sequences, but others do not). By extending the fragment size, the effect caused by fragmentation can be reduced, and the comparison between genomes will become easier. In Fig. 1 Ci, the fragmentation window size is set to 1 Mb, and the window is moved in 100-kb steps to reduce the effects of the 1-Mb cutting positions. The number of sequences used for BLSOM is almost the same as in the analysis shown in Fig. 1Bi, and only the effects of increasing the fragmentation size can be detected; the number of black nodes is decreased, and the separation between species becomes clearer (Fig. 1 Ci). The human and bat genomes show clear separation, but for the genomes of both species, there is a broad horizontal distribution and subdivision of each species territory (Fig. 1Cii), which seems to be related to mosaic G + C% structures typically observed in higher vertebrates [13, 26, 27]. Since the separation pattern is simpler than that under 100-kb fragmentation (Fig. 1B), 1-Mb fragmentation is used in the following analyses.

The BLSOM is equipped with a tool known as the U-matrix [28], which displays the Euclidean distances between representative vectors of neighboring nodes as the degree of blackness; a larger distance is reflected by a higher degree of blackness (Fig. 1Ciii). A distinct black zone is observed in the upper right part of the human territory, indicating that the oligonucleotide compositions of the sequences located in that region not only differ distinctly from the majority of human sequences but also differ from each other. In our previous BLSOM analysis of the human genome, which mainly focused on tetra- to hexanucleotides, we found a similar conspicuous black zone of the U-matrix; we called this zone the “specific zone” (Sz), and we reported that the sequences derived from centromeric and pericentromeric heterochromatin regions were clustered there [7, 14–16]. In the present study, the similar black region identified by the U-matrix is also referred to as Sz. The BLSOM is an explainable-type machine learning algorithm that reveals why sequences have been separated (self-organized) by using a red/blue heatmap (Fig. 1D) [20–22]; the contribution level of each oligonucleotide at each node can be visualized based on color: red (high), white (moderate) and blue (low). Interestingly, GA + TC shows a distinctive red/blue distribution that stands out from that of other dinucleotides, with a major dark red area in Sz; more specifically, the occurrence frequency is low (blue in Fig. 1D) in most regions, including the bat territories, but evident enrichment (dark red) can be seen in the majority of Sz. Importantly, this is not a property derived from the mononucleotide composition because the distribution is very different from that of AG + CT, which has the same mononucleotide composition. Some enrichment (though not as conspicuous as that of GA + TC) of other oligonucleotides (e.g., AC + GT) is also observed in Sz.

### DegeDinucleotide distribution on human chromosomes

To investigate the findings obtained with the BLSOM in detail, we next use a more direct analysis method, a standard distribution map. First, to test the local evident enrichment of GA + TC in the human genome, which can be predicted from the dark red portion of Sz, we examined the distribution of the occurrence frequency (%) of the dinucleotide on each human chromosome, as well as AC + GT for comparison, in 1-Mb sliding windows with a 100-kb step size. Figure 1E provides examples of two autosomal chromosomes (metacentric chr1 and acrocentric chr14) and two sex chromosomes (chrX and Y). The results for the other chromosomes are shown in Supplemental Fig. S1. Human centromeric and pericentromeric heterochromatin regions are composed of highly repetitive sequences and are incompletely sequenced even in the currently available GRCh38/hg38 version; the unsequenced region is visualized as an open space in Fig. 1E, where the central position of the primary constriction of each chromosome shown on the UCSC Genome Brower (https://genome.ucsc.edu/) is indicated by the vertical magenta bar, and the genomic positions of centromeric and pericentromeric heterochromatin regions are presented in Supplemental Table S2. Notably, on chr1, there is a large constitutive heterochromatin region (1q12) adjacent to the centromeric region on the long arm (UCSC genome browser, https://genome.ucsc.edu/), and this region has not been sequenced; on acrocentric chr14, the large heterochromatin region on the centromeric side is also unsequenced and is thus left blank.

Interestingly, GA + TC (brown circles) was clearly enriched in centromeric and pericentromeric regions of not only the chromosomes shown here but all chromosomes (Supplemental Fig. S1), showing very peculiar oligonucleotide compositions in these regions. These findings are consistent with our previous finding that Sz in the U-matrix is composed of sequences from centromeric and pericentromeric heterochromatin regions [14–16]. AC + GT (blue circles) was enriched in the centromeric and pericentromeric regions of only some chromosomes, and this enrichment was less evident than that of GA + TC. Furthermore, on many chromosomes, AC + GT tended to be lacking in the regions of interest.

### CG distribution on bat scaffolds

The genome G + C% is slightly over 40% for both humans and bats [29], and the number of sequences enriched in C and G is therefore small. The red zone in Fig. 1D for dinucleotides consisting of only C or G is restricted to the left side, and the CC + GG content is high (red) in a somewhat broadened area on the left side, but the GC content is high in the middle to lower part of the left side. However, the CG content is high only in the lower part on the left side and extends significantly into the Aeg territory; some other bat sequences are also located around this red region. Considering CG suppression (i.e., CG deficiency) [30], which is typically observed in mammalian genomes and is thought to be related to the C-methylation of CG, the local enrichment in CG in bat territories, especially in the Aeg territory (Fig. 1D), is interesting. We then examined the distribution of the CG frequency in bat genomes to clarify the chromosomal locations of CG-enriched sequences. Figure 1F shows the distribution of the CG composition (%) in 1-Mb fragments with a 100-kb sliding step for the long scaffolds of three bats. The most distinct CG peaks were observed at both ends of the longest and second longest scaffolds (Scaf1 and 2) of Aeg. Since only long scaffolds were used in the distribution analysis, these are likely to correspond to chromosomes, but they are called scaffolds according to the original paper [10]. In the long scaffolds of other bat species, distinct peaks were also observed, at least at one end (Fig. 1F and Supplemental Fig. S2), but the degree of enrichment was less than that in Aeg, which is consistent with the heatmap results in Fig. 1D. Since the evident peak was composed of data from many 1-Mb sequences, it appears to be appropriate to call it an “Mb-level CpG island”. It should also be noted that in regions other than the scaffold end, Fer shows distinct internal peaks, which will be discussed later.

CpG islands, which play crucial roles in transcriptional regulation, are typically a few hundred bp in length and preferentially occur in gene regulatory regions [31]. By zooming out from the hundred-bp level to the Mb level, conventional CpG islands become inconspicuous, and Mb-level peaks become prominent in bat genomes. In view of the biological importance of CpG islands, we refer to these large-scale structures as “Mb-level CpG islands” [15]. Notably, in a wide range of vertebrate genomes, including the human genome, it has long been established that the G + C% tends to be higher towards the ends of the chromosomes [26]. If we use the term CpG island, we should verify that the Mb-level CG enrichment is not a mere reflection of increase in the G + C%. By analyzing the distribution of the odds ratio (Obs/Exp) of CG, which is obtained by dividing the observed occurrence of CG the its expected value according to the mononucleotide composition, evident peaks were observed in the terminal region of the long scaffolds of bats (Supplemental Fig. S3). It was therefore appropriate to call the peaks “Mb-level CpG islands”.

### DegeTri-BLSOM of three bats and humans

**Fig. 2:**
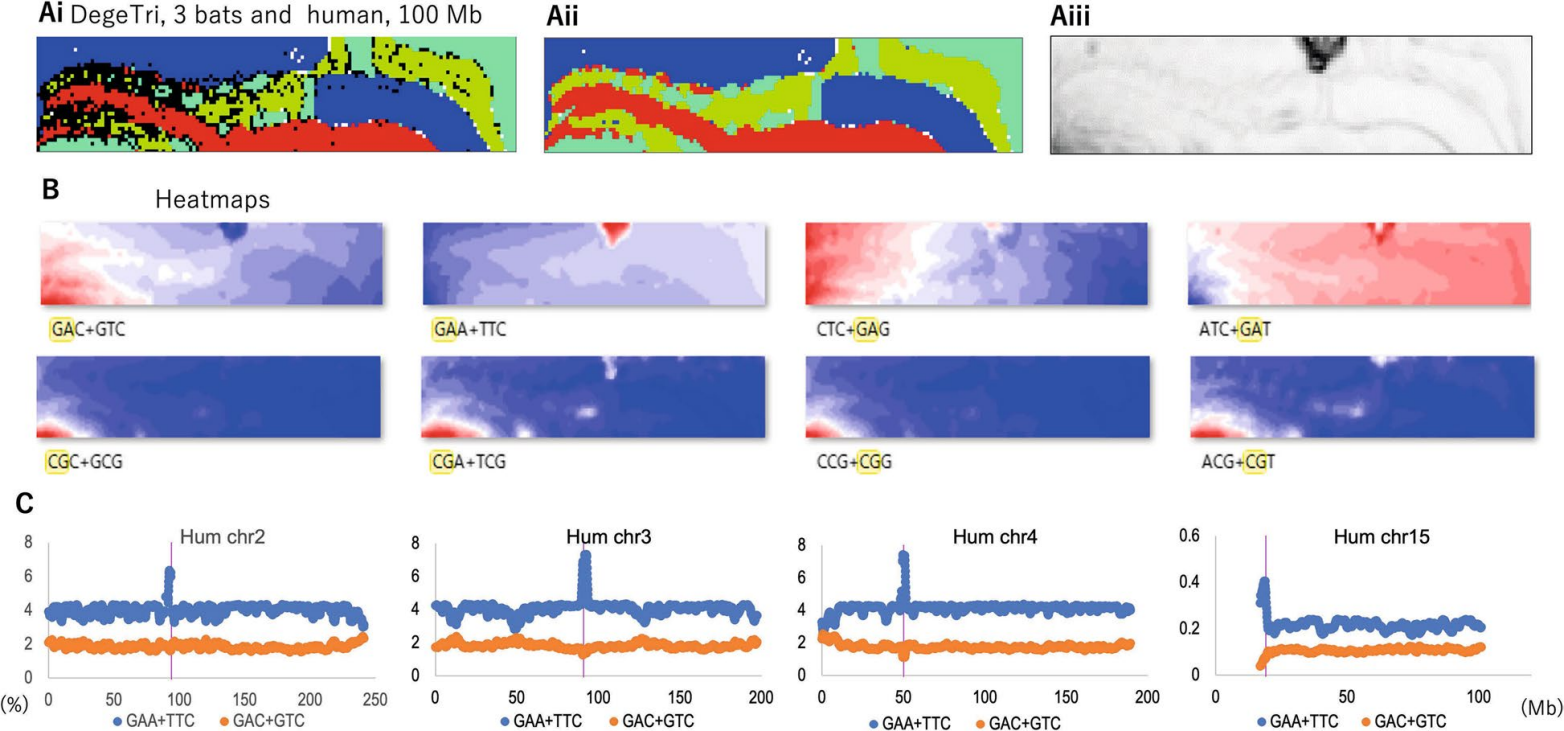
DegeTri-BLSOM for 3 bats and humans and the distribution of the DegeTri frequency on human chromosomes. **Ai** The DegeTri-BLSOM for 1-Mb sequences with a 100-kb sliding step for 3 bats and humans was constructed and displayed as described in Fig. 1B. **Aii** Each grid point is colored according to the species with the highest number of sequences. **Aiii** U-matrix. **B** Heatmaps of GA- or CG-containing DegeTri: upper or lower panels, respectively. **C** The occurrence frequencies (%) of (GAA + TTC) and (GAC + GTC) plotted on four human chromosomes as described in Fig. 1E. Chr15 is an example of an acrocentric chromosome

Considering the biological significance of the Mb-level peaks obtained from the dinucleotide analysis, we next examined whether the Mb-level enrichment represents the properties of the dinucleotide itself or reflects the properties of longer oligonucleotides. Figure 2A shows the BLSOM with the degenerate trinucleotide (DegeTri) compositions of humans and the three bats. The upper panels in Fig. 2B show the heatmap of the DegeTri containing GA (and, thus, TC). The heatmaps of all DegeTris (Supplemental Fig. S4) also show that the addition of a different nucleotide to GA + TC produces different effects (Fig. 2B). This shows that the specific enrichment of GA + TC observed in Fig. 1D is a reflection of a longer oligonucleotide rather than the dinucleotide itself. The lower panels in Fig. 2B show the heatmap of the DegeTri containing CG. The prominent small red region in the lower left portion of the bat region is fairly unaffected by the type of nucleotide added to CG. In other words, the prominent small red region reflects the characteristics of the distribution of the CG dinucleotide itself in the bat genome. In the case of Sz in the human territory, the addition of A or T (but not of C or G) results in a slight increasing trend (from blue to white in the heatmaps).

Figure 2C shows the distribution of GAA + TTC and GAC + GTC, which are examples of the addition of one different nucleotide to GA + TC. In this figure, we purposely chose different chromosomes from those in Fig. 1E to present the results of diverse chromosomes. As also shown in Supplemental Fig. S5, in which the results for all human chromosomes are presented, GAA + TTC (blue circles) is clearly enriched in centromeric and pericentromeric regions of all chromosomes, whereas GAC + GTC (brown circles) tends to be scarce in these regions. This shows that the specific enrichment of GA + TC observed in Fig. 1D is a reflection of longer oligonucleotides rather than the dinucleotide itself.

### Analyses of DegePenta for three bats and humans

**Fig. 3:**
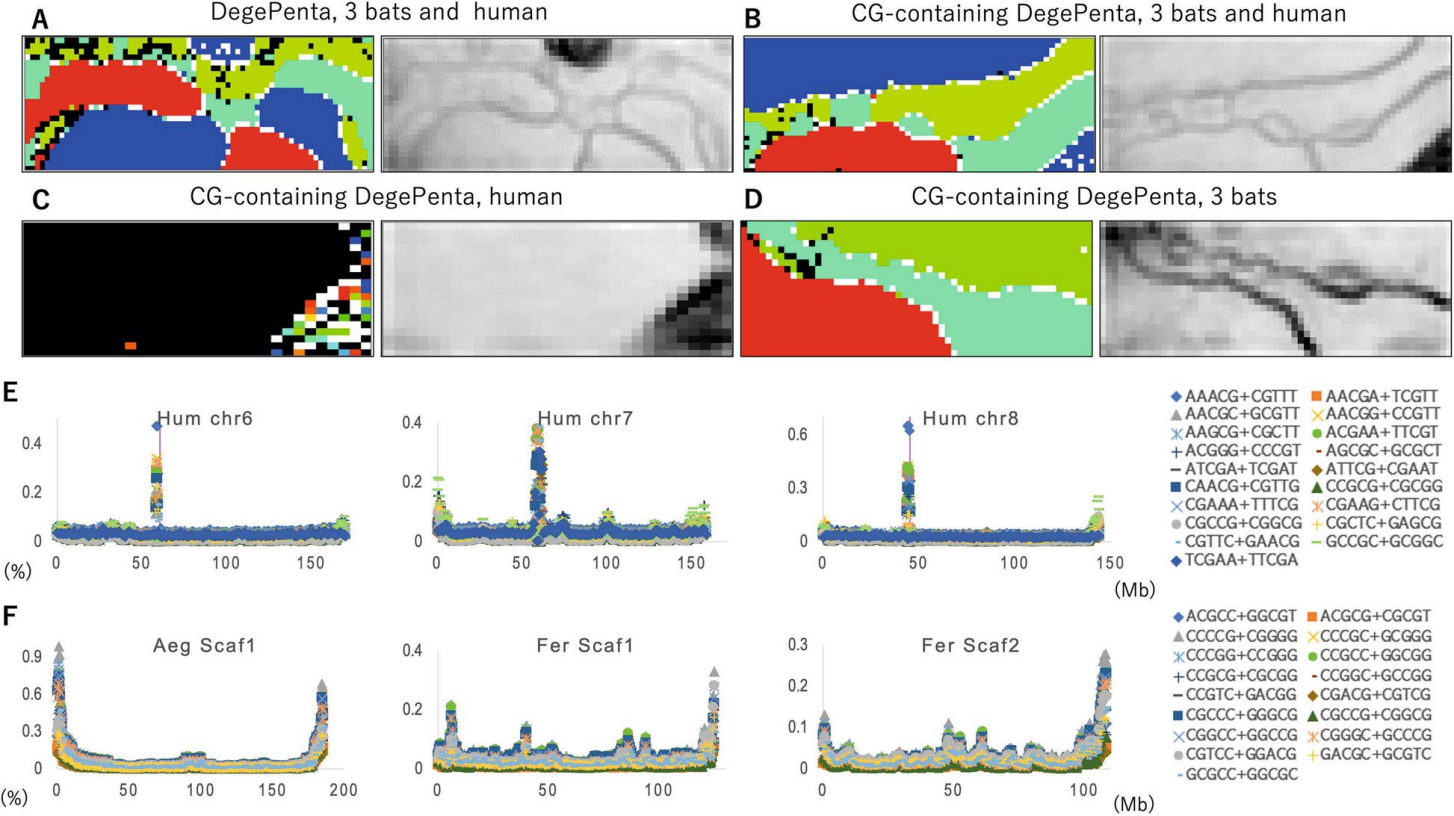
DegePenta-BLSOMs for 3 bats and humans and the distribution of the DegePenta frequency on their chromosomes. **A** DegePenta-BLSOM for 1-Mb sequences with a 500-kb sliding step for 3 bats and humans and its U-matrix. In the case of DegeDi- and DegeTri-BLSOMs, the 1-Mb window was slid in 100-kb steps for comparison with the results of 100-kb fragmentation. However, to reduce only the effects of the cutting positions of the 1-Mb window, the 100-kb step is too narrow and consumes considerable computation time in high-dimensional analyses. Therefore, the step size was changed to 500 kb. **B** CG-containing DegePenta-BLSOM for 3 bats and humans and its U-matrix. **C** CG-containing DegePenta-BLSOM for humans and its U-matrix. In the BLSOM, nodes containing sequences from more than one chromosome are indicated in black, and those containing sequences from a single chromosome are indicated in chromosome-specific colors; for the meanings of the colors, see Supplemental Table S2. **D** CG-containing DegePenta-BLSOM for 3 bats and its U-matrix. **E** The occurrence frequency (%) of the top ten DegePenta on three human chromosomes is displayed as described in Fig. 1E. On the three chromosomes, the top ten oligonucleotides differed from each other, but for each oligonucleotide, the common symbols presented in the rightmost panel are used for the three chromosomes. The (Max-Ave)/Ave ratio was used for the top ten selections, but the occurrence frequency (%) itself is displayed here. **F** The occurrence frequency (%) of the top ten DegePentas on three bat scaffolds is displayed. The common symbols are used, independent of the scaffold

By analyzing tetra- or pentanucleotides, relationships with biological functions (e.g., binding to proteins) can be examined more directly. Figure 3A shows the DegePenta-BLSOM of 1-Mb sequences with a 500-kb sliding step and the corresponding U-matrix. The pattern is simpler than that of DegeTri, and the number of black nodes is reduced, which may provide a more accurate understanding of the characteristics of each genome. However, since we are dealing with 512 variables, it is not easy to investigate the relationships with biological functions by analyzing the 512 heatmaps. In the following analyses, we therefore attempt to focus on oligonucleotides, which are thought to be important from the perspective of biological functions. Figure 3B shows the BLSOM with CG-containing DegePentas for the three bats and humans. Here, a total of 122 variables are used, the separation according to species becomes clearer, and the number of black nodes is reduced relative to the DegePenta-BLSOM with 512 variables. For human territories, the region judged to represent Sz by the U-matrix is well separated from the human main territory by several bat territories, showing that the CG-containing DegePenta composition of Sz sequences should differ markedly from that of other human sequences. The heatmaps presented in Supplemental Fig. S6 show that a portion of the CG-containing oligonucleotides are enriched in a limited or wide area in Sz, and others are distributed rather evenly over a broad area in human and/or bat territories. Since these results are still complex, it appears to be convenient to first analyze the human and bat genomes separately and then compare them.

### Analyses of CG-containing DegePentas in humans

In Fig. 3C, only human genome sequences are analyzed. Here, nodes that contain a mixture of sequences from different chromosomes are shown in black, and nodes that contain only sequences from a single chromosome are shown in chromosome-specific colors. Interestingly, in the Sz area in this BLSOM, there are a large number of colored nodes showing chromosome-dependent separation, but there is practically no separation in the major territory (black nodes). In addition, there are blank (colorless) nodes in Sz that do not contain any sequences. When sequences whose compositions are clearly distinct from those of other sequences exist, no sequences are attributed to the nodes adjacent to those of the distinct sequences after learning, resulting in blank nodes [14–16]. This shows that the sequences in Sz should distinctly differ in their oligonucleotide composition not only from a majority of other human sequences but also from each other. Our previous distribution analysis of CG-containing oligonucleotides on human chromosomes [15, 16] showed that when the sequence surrounding CG is composed primarily of A or T, the oligonucleotides are enriched in centromeric and pericentromeric heterochromatin regions, but if the sequences surrounding CG are composed primarily of G or C, the oligonucleotides tend to also be enriched in subtelomeric regions. The same tendency was of course observed in the present analysis, but when all 122 CG-containing DegePentas were analyzed in detail, stricter sequence dependency that could not be explained merely by the composition of the surrounding mononucleotides was observed. Notably, only one-third of the CG-containing oligonucleotides were specifically enriched in Sz, and the patterns often differed completely even among highly similar oligonucleotide sequences (see heatmaps of Supplemental Fig. S6). This finding may be related, at least in part, to the presence of many TFBSs that contain the CG sequence, as discussed below.

To clarify the enrichment of CG-containing DegePentas in Sz via conventional distribution analysis, we analyzed the distribution on each human chromosome as follows. First, the occurrence frequency (%) of each oligonucleotide in 1-Mb sequences was calculated across each chromosome, and oligonucleotides with high (Max-Ave)/Ave values were selected, where Ave is the average occurrence value of each oligonucleotide on each chromosome, and Max is the highest value for the oligonucleotide among all 1-Mb sequences on the chromosome. Division by the average value allowed us to exclude the case in which the high peak was a mere reflection of a high basal level across the chromosome. After sorting the obtained ratios, we examined the distribution of the occurrence frequency (%) of the top 10 oligonucleotides on each chromosome. Figure 3E shows examples of three chromosomes with moderate chromosome lengths (chr6-8), and Supplemental Fig. S7 shows all chromosomes. Interestingly, for all chromosomes, the top 10 oligonucleotides were enriched in centromeric and pericentromeric regions, and slight increases were observed in the subtelomeric region. In Fig. 3E, common symbols are used for different chromosomes, and it is therefore clear that the set of oligonucleotides included in the highest peak differs among chromosomes. This shows that the combination of enriched oligonucleotides in centromeric and pericentromeric regions clearly depends on the chromosome, and this is the reason for the existence of many colored nodes in Fig. 3C.

Methylation at C in CG dinucleotides is a typical epigenetic modification, and the binding of methyl-CpG-binding domain proteins (MBDs) as well as several structurally unrelated methyl-CpG-binding zinc-finger proteins to methylated C bases induces histone deacetylation, subsequent chromatin condensation and heterochromatinization [32]. The human methyl-CpG-binding protein MeCP2 requires an A/T-rich sequence surrounding the methylated C for its binding and is involved in the formation of chromatin loops and nuclear organization [30, 32]. The chromosome-dependent enrichment of CG-containing oligonucleotides in centromeric and pericentromeric regions may be related to the differential sequence specificity of methyl-CpG-binding proteins.

### Analyses of CG-containing DegePentas in three bats

The CG-containing DegePenta-BLSOM for the three bats is shown in Fig. 3D. The separation by species is clear, indicating that the genomic composition of CG-containing DegePentas clearly differs by species. Figure 3F shows the distribution of the top ten oligonucleotides according to the (Max-Ave)/Ave ratio on long scaffolds. As observed in the CG distribution in Fig. 1F, Aeg shows a pronounced Mb-level peak at both ends and low peaks in the interior region. For Fer, the terminal peak is reduced in height to 30% of the level in Aeg, and clear internal peaks are observed, which is consistent with the results in Fig. 1F. To illustrate that these internal peaks are not limited to a specific scaffold, Fig. 3F shows the pattern of the second longest scaffold (Scaf2) for Fer, in which distinct internal peaks are also observed. In Supplemental Fig. S8, we show examples of the three longest scaffolds of all three bats, and evident terminal peaks and internal peaks are observed, although their height and thickness vary among species. It should also be noted that we have previously observed Mb-level CpG islands near the ends of frog chromosomes [33], and the evolutionary processes that formed the Mb-level CpG islands are therefore of interest.

### DegeHexa-BLSOM and analyses of human DegeHexa TFBSs

**Fig. 4:**
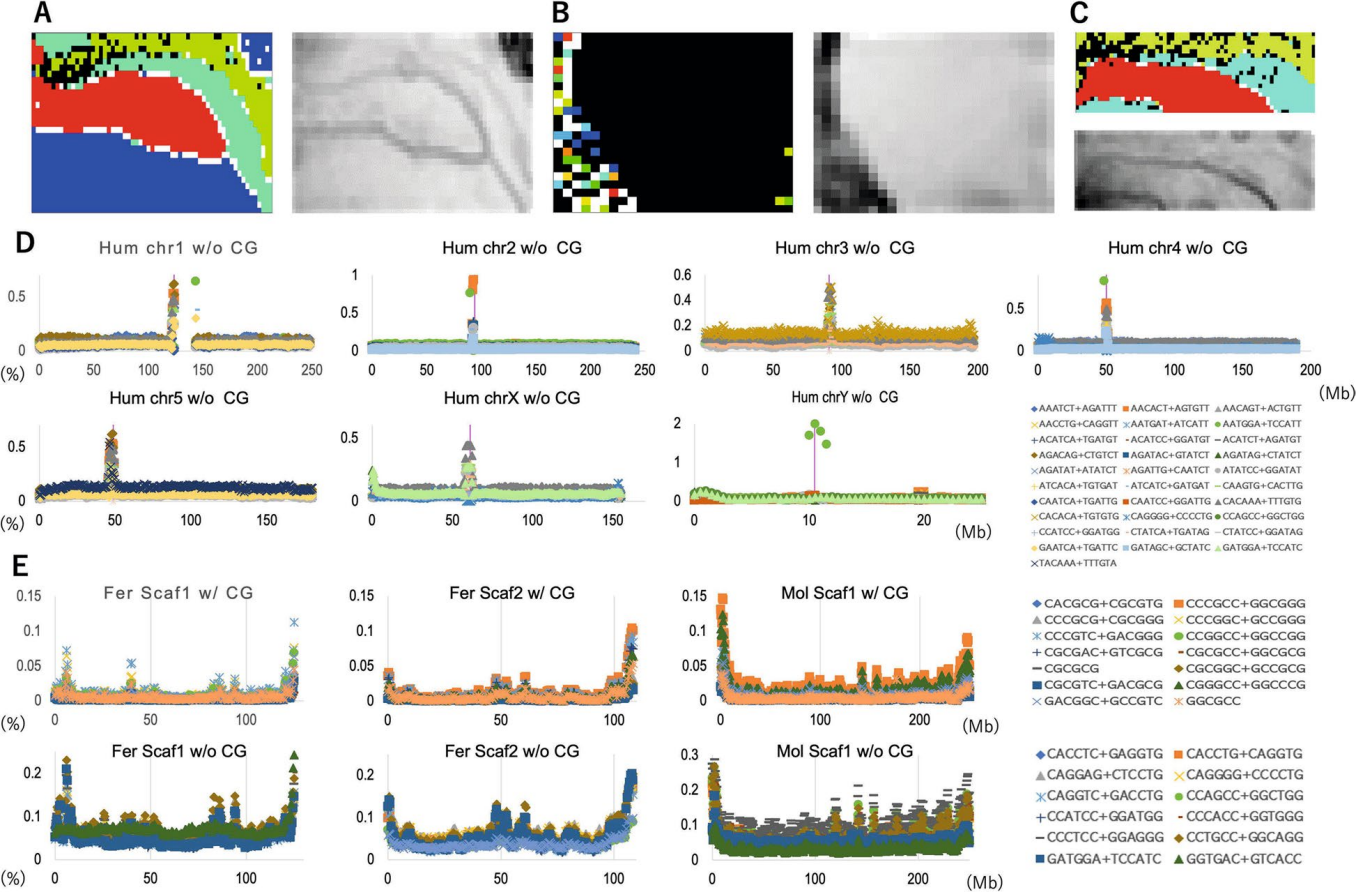
DegeHexa- and TFBS-DegeHexa-BLSOMs for 3 bats and humans and the distribution of the TFBS-DegeHexa frequency on their chromosomes. **A** DegeHexa-BLSOM for 1-Mb sequences with a 500-kb sliding step for 3 bats and humans and its U-matrix. **B** TFBS-DegeHexa-BLSOM for humans and its U-matrix. Nodes are displayed as described in Fig. 3C. **C** TFBS-DegeHexa-BLSOM for 3 bats and its U-matrix. **D** The occurrence frequencies (%) of the top ten DegeHexa TFBSs on seven human chromosomes is displayed. The common symbols (listed in the last panel) are used, regardless of the chromosome. **E** The occurrence frequencies (%) of the top ten DegeHexa TFBSs with (w/) or without (w/o) CG on three scaffolds are displayed in the upper and lower panels, respectively. To facilitate the comparison of their peak positions, vertical lines of auxiliary scales are included. The common symbols (listed in the rightmost panel) are used, independent of the scaffold

The DegeHexa-BLSOM of 1-Mb sequences with a 500-kb sliding step for the three bats and humans is shown in Fig. 4A; the species-dependent separation pattern is clearer than that of DegePenta-BLSOM in Fig. 3A. The human territory is largely divided into the main territory and the Sz territory defined by the U-matrix, and these two territories are separated by several bat territories, showing that the DegeHexa composition in Sz clearly differs from that in the main human territory. Since DegeHexa consists of 2080 variables, knowledge discovery from 2080 heatmaps is quite difficult, and we adopted the following strategy to investigate the relationships with biological functions. In the case of hexanucleotides, many TFBSs have been assigned to the human genome by the SwissRegulon Portal [34]. Transcription factors (TFs) are evolutionarily well preserved due to their functional importance, and a large number of human TFBSs are thought to also be used in bat genomes. Hence, we initially performed a BLSOM analysis of 181 TFBSs in the human genome (Fig. 4B), that were assigned to the human genome by the SwissRegulon Portal (for the TFBS sequences, see Supplemental Table S1). The nodes are colored according to the chromosome; in Sz, there are many colored and, thus, chromosomally separated nodes, showing that the TFBS composition in Sz sequences differs by chromosome.

Next, we analyzed the TFBS distribution on each chromosome. When searching for TFBSs with a high (Max-Ave)/Ave ratio, we observed that CG-containing TFBSs often presented a high ratio, making it difficult to distinguish the effect of a TFBS from that of CG alone. Therefore, in Fig. 4D, the top ten TFBSs that did not contain CG (w/o CG) are shown for five autosomes (chr1-5) and two sex chromosomes (in Supplemental Fig. S9, distribution maps of the top 10 TFBSs are presented regardless of the presence or absence of CG for all chromosomes). Importantly, on all chromosomes, distinct peaks were observed in centromeric and pericentromeric regions (Fig. 4D and Supplemental Fig. S9), representing “Mb-level TFBS islands”. As shown in Fig. 4D, chrY, on which a specific AATGGA+TCCATT sequence is evidently enriched, clearly differs from the other chromosomes. By analyzing both hexa- and heptanucleotide TFBSs [16], we previously showed that “Mb-level TFBS islands” are located in centromeric and pericentromeric constitutive heterochromatin regions, as reported by Strachan and Read (2004) [35] and the UCSC genome browser (https://genome.ucsc.edu/). The positions of Sz sequences obtained from the BLSOM shown in Fig. 4B were found to be almost the same as those of centromeric and pericentromeric constitutive heterochromatin regions (Supplemental Table S2). Since all Sz sequences are derived from centromeric and pericentromeric heterochromatin regions, we could estimate the number of DegeHexa TFBSs enriched in these genomic regions simply by examining the red/blue heatmap levels in Sz in the BLSOM presented in Fig. 4B, and we found that approximately 100 out of 181 TFBSs showed a distinct red color in Sz (Supplemental Fig. S11). This showed Mb-level enrichment in these regions for more than half of the TFBSs; some TFBSs were red in a large area in Sz (enrichment for many chromosomes), and others were locally red (enrichment for a limited number of chromosomes).

### Analyses of TFBS-containing DegeHexa in 3 bats

In Fig. 4C, we show the BLSOM of three bats for 181 DegeHexa TFBSs assigned to the human genome. Although the separation of the microbat Myo from those of other bats is clear, there is significant overlap between the microbat Fer and the megabat Aeg, which are closely related according to molecular phylogeny; most of the black nodes were found to be a mixture of the last two sequences (data not shown). We then analyzed the distribution of the DegeHexa-TFBS frequency (per Mb) across long scaffolds to examine whether and where TFBS peaks exist in bat genomes (Fig. 4E). TFBSs were again sorted according to the (Max-Ave)/Ave ratio, and the top 10 TFBSs with and without CG are presented in the upper and lower panels, respectively. In the longest scaffold (Scaf1) of Fer, distinct peaks were observed for TFBSs containing CG (upper panel of Fig. 4E), and the internal peaks were located at almost the same positions for TFBSs without CG (lower panel of Fig. 4E), although peak heights differed from each other. Similar results were observed for Fer scaffold 2 (Scaf2). For Aeg, the internal peaks of CG-containing TFBSs (Supplemental Fig. S8) were rather inconspicuous because of the evidently high occurrence of peaks at both ends. When the peak positions were examined with different vertical scales, the positions of these internal peaks were almost the same as those of TFBSs without CG (lower panel). In addition, we present an example from Mol, which showed relatively low terminal peaks and easily visible internal peaks. The results for the longest 3 scaffolds of all six bats are presented in Supplemental Fig. S10. The finding that various TFBSs with or without CG form peaks at almost the same positions in long bat scaffolds shows that Mb-level structures enriched in diverse TFBSs exist at internal chromosomal sites not only in the human genome but also in bat genomes, although the enrichment level in the bat genomes is clearly lower than that in the human genome.

### Biological significance of Mb-level TFBSs and CpG islands

In this comparative genomic study, without setting up models or hypotheses, a main part of knowledge discovery was left to machine learning, especially at the beginning of the analysis. The most characteristic result obtained in this data-driven study was the remarkable enrichment of TFBSs in human centromeric and pericentromeric regions, which raises various questions for us. Although such issues appear to be somewhat outside the scope of the comparative genomic study, we will briefly discuss the possible function of Mb-level TFBS islands for which sufficient knowledge has accumulated on the human genome side. By analyzing Hi-C data for Mb-level interchromosomal interactions published by Lieberman-Aiden E, et al. [17], we previously found that chromatin segments supporting Mb-level interchromosomal interactions were primarily located in Mb-level TFBS islands [16], and we therefore proposed that TFs and thus TFBSs are important in centromere clustering and thus large-scale formation of the 3D architecture of genomic DNAs in interphase nuclei. Recently, the functions of TFs beyond their known gene expression-regulatory functions, such as the induction of chromatin looping, changing chromatin and nuclear structure, have received much attention [36, 37]. Among these functions, TF-mediated looping interactions between two different genomic regions has attracted wide attention [38–40]. Mb-level TFBS and CpG islands may be involved in the large-scale nuclear organization as explained in detail below, and the role of Mb-level CpG islands found near the ends of bat chromosomes in the nuclear arrangement of bat genomic DNAs is particularly interesting.

A well-known function of pericentromeric regions is the formation of condensed heterochromatin in chromocenters, which supports the association of centromeric and pericentromeric DNAs of homologous and nonhomologous chromosomes [40–43, 44], and the number and size of chromocenters, as well as the number and type of centromeres gathered in each chromocenter, vary depending on cell type; in addition, telomeric regions are associated as the chromocenter grows larger [43]. The chromosome-dependent enrichment of a combination of TFBSs as well as CG-containing oligonucleotides in centromeric and pericentromeric regions is thought to be involved in supporting the cell type-dependent centromere clustering because the cellular contents of individual TFs as well as the levels of CG methylation enzymes and methyl-CpG-binding proteins are regulated in a cell type-dependent manner.

Considering these accumulated findings about the human genome, the Mb-level structures found in the bat genome may also be involved in the formation of nuclear 3D structures, and future analysis of Hi-C data for Mb-level interchromosomal interaction for the bat genome will clarify the function of Mb-level CpG islands locating near telomeres. It should also be mentioned that we have recently analyzed short oligonucleotide compositions of a wide range of coronaviruses isolated from bats or humans and found that levels of CG were noticeably higher in viruses from bats (our unpublished results). The levels of CG in the host genome and mRNAs were also higher on the bat side, which is interesting when considering the biological significance of CG in bat hosts and bat-derived viruses (should be published elsewhere).

To consider the molecular mechanisms underlying the functions of Mb-level structures in more detail, we must know the detailed sequence characteristics of the Mb-level islands. Our preliminary sequence-level analyses of Mb-level TFBS islands [16] indicated that one reason for the observed chromosome-dependent enrichment is related to the chromosome-dependent difference in alpha-satellite monomer sequences [45–47]. Notably, the hexanucleotide composition of the consensus sequence of alpha-satellite sequences has been analyzed by several groups, and it has been reported that the most evident sequence is GAAACA, while the others are AGAAAC, GAGCAG, AAACAC and AGAGAA [48, 49]. None of the five sequences found within the consensus sequence or their complementary sequences are included in the hexanucleotide TFBSs that were reported by the SwissRegulon Portal and, thus, used in the present study. Collectively, the results show that the enrichment of TFBSs at and near the centromeres is chromosome dependent and is not a feature of the consensus sequence of alpha-satellite sequences.

### Future prospects

In the Background section, we mentioned that one of the goals of the comparative genomic analysis of the bat and human genomes is to aid future research on the molecular evolution of various zoonotic infectious disease-causing viruses after invading the human population. The present study has not yet been linked to viral evolutionary studies and is limited to a comparative study of the host genomes themselves. Recently, an informatics search for TFBSs that are present in the SARS-CoV-2 genome but absent in the bat coronavirus genome identified 22 TFBSs that are presumed to facilitate viral replication [50]. All of these TFBSs are longer than hexanucleotides, and their relationship to the TFBSs included in the present analysis is therefore unclear. When we extend our analysis to TFBSs with longer sequences in various zoonotic viruses, we can perform a detailed characterization of the TFBSs that are absent in the bat-derived strains but present in the human-derived strains and whose occurrence increases in the viral genome during human-to-human transmission after invading the human population, and we are planning to conduct such research as a separate project.

## Conclusion

Combining the oligonucleotide BLSOM and the distribution map method, we conducted a comparative genome study of humans and bats and found the Mb-level enrichment of CG (Mb-level CpG islands) around the termini of bat long-scaffold sequences and, thus, most likely in subtelomeric regions. We also found the chromosome-dependent Mb-level enrichment of a set of TFBSs (Mb-level TFBS islands) in centromeric and pericentromeric heterochromatin regions of human chromosomes. The Mb-level TFBS and CpG islands are thought to be involved in the large-scale nuclear organization, such as centromere and telomere clustering. Our machine learning-based analysis will help us to understand the differential features of nuclear 3D structures in the human and bat genomes.

The phylogenetic approach based on sequence alignment is a well-established and irreplaceable method for comparative genomic and molecular evolutionary studies [51]. Notably, the sequence alignment-free method used in this study is suitable for analyzing a large amount of sequence data [7, 21]. Furthermore, unsupervised machine learning can be used without special models or presumptions and has powerful visualization capabilities enabling efficient, novel knowledge discovery from big sequence data. Genomic and evolutionary studies have entered the era of big data, and AI technologies including machine learning methods can complement conventional phylogenetic methods, especially when a massive number of sequences are being considered.

## Supplementary Information

**Additional file 1: Supplemental Fig. S1. **Distribution map for AC+GT and GA+TC on all human chromosomes.**Supplemental Fig. S2.** Distribution map for CG on longest 3 scaffolds of 6 bats.**Supplemental Fig. S3.** Distribution map for Odds for CG on longest 3 scaffolds of 3 bats.**Supplemental Fig. S4. **Heatmaps of DegeTri BLSOM presented in Fig. 2A.**Supplemental Fig. S5. **Distribution map for GAA+TTC and GAC+GTC on all human chromosomes.**Supplemental Fig. S6. **Heatmaps of CG-containing DegePenta BLSOM presented in Fig. 3B.**Supplemental Fig. S7. **Distribution map for top 10 of CG-containing DegePenta on all human chromosomes.**Supplemental Fig. S8. **Distribution map for top 10 of CG-containing DegePenta on longest three scaffolds of 3 bats.**Supplemental Fig. S9. **Distribution map for top 10 of DegeHexa TFBSs on all human chromosomes.**Supplemental Fig. S10. **Distribution map for top 10 of DegeHexa TFBSs on longest 3 scaffolds of 6 bats.**Supplemental Fig. S11. **Heatmaps of human DegeHexa-TFBS BLSOM presented in Fig. 4B.

**Additional file 2: Supplemental Table S1.** Human DegeHexa TFBS sequences. **Supplemental Table S2.** List of Sz regions in each human chromosome.

## Data Availability

All genomics sequences used in this study are available at UCSC ftp site (http://hgdownload.soe.ucsc.edu/goldenPath/hg38/chromosomes/) and https://bds.mpi-cbg.de/hillerlab/Bat1KPilotProject/.

## Abbreviations

AI: Artificial intelligence
SOM: Self-organizing map
BLSOM: Batch-learning self-organizing map
TFBS: Transcription factor binding sequence
Hi-C: Chromatin conformation capture
DegeDi: Degenerate dinucleotide
DegeTri: Degenerate trinucleotide
DegePenta: Degenerate pentanucleotide
DegeHexa: Degenerate hexanucleotide
Sz: Specific zone
Aeg: *Rousettus aegyptiacus*
Fer: *Rhinolophus ferrumequinum*
Myo: *Myotis myotis*
Dis: *Phyllostomus discolor*
Kuh: *Pipistrellus kuhlii*
Mol: *Molossus molossus*
Scaf1: The longest scaffold
Scaf2: The second longest scaffold
