# Supplementary figures and images for: Comparative genomic analysis of the human genome and six bat genomes using unsupervised machine learning: Mb-level CpG and TFBS islands
Source: BMC Genomics. 2022 Jul 8;23:497. doi: 10.1186/s12864-022-08664-9 (PMC9264310; doi:10.1186/s12864-022-08664-9)

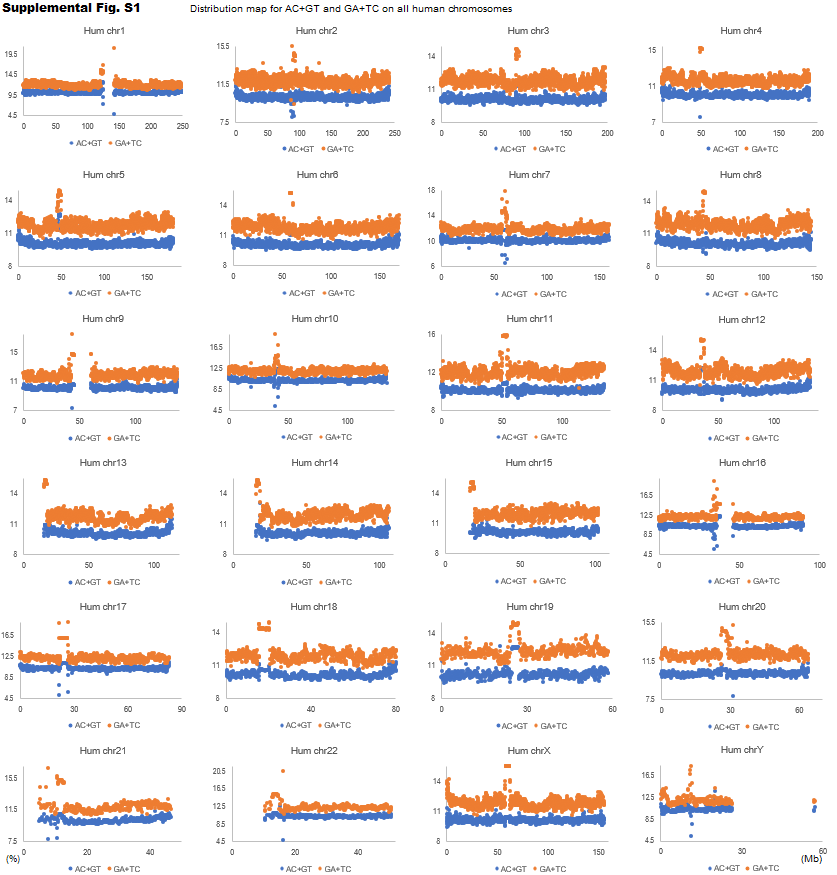


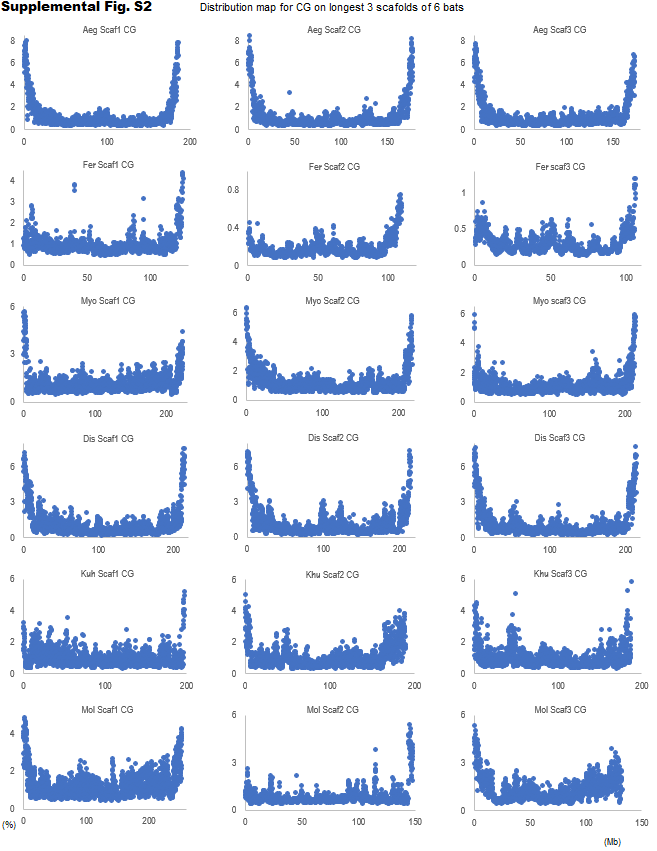


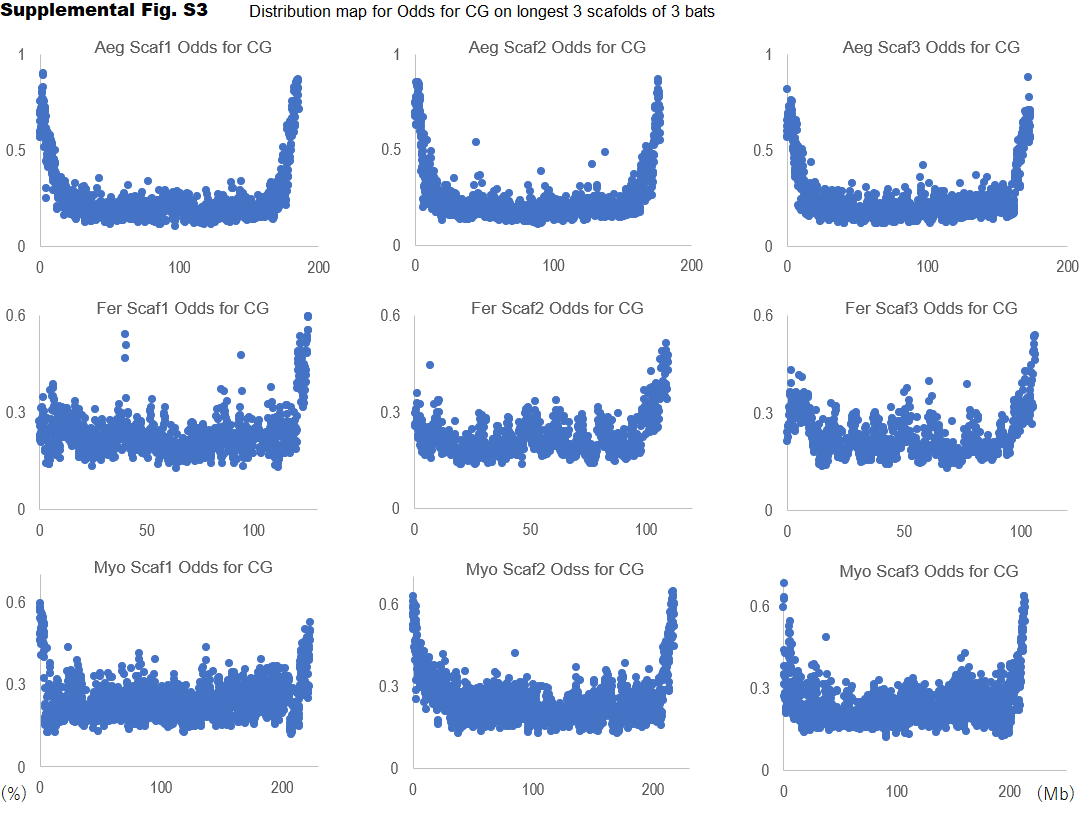


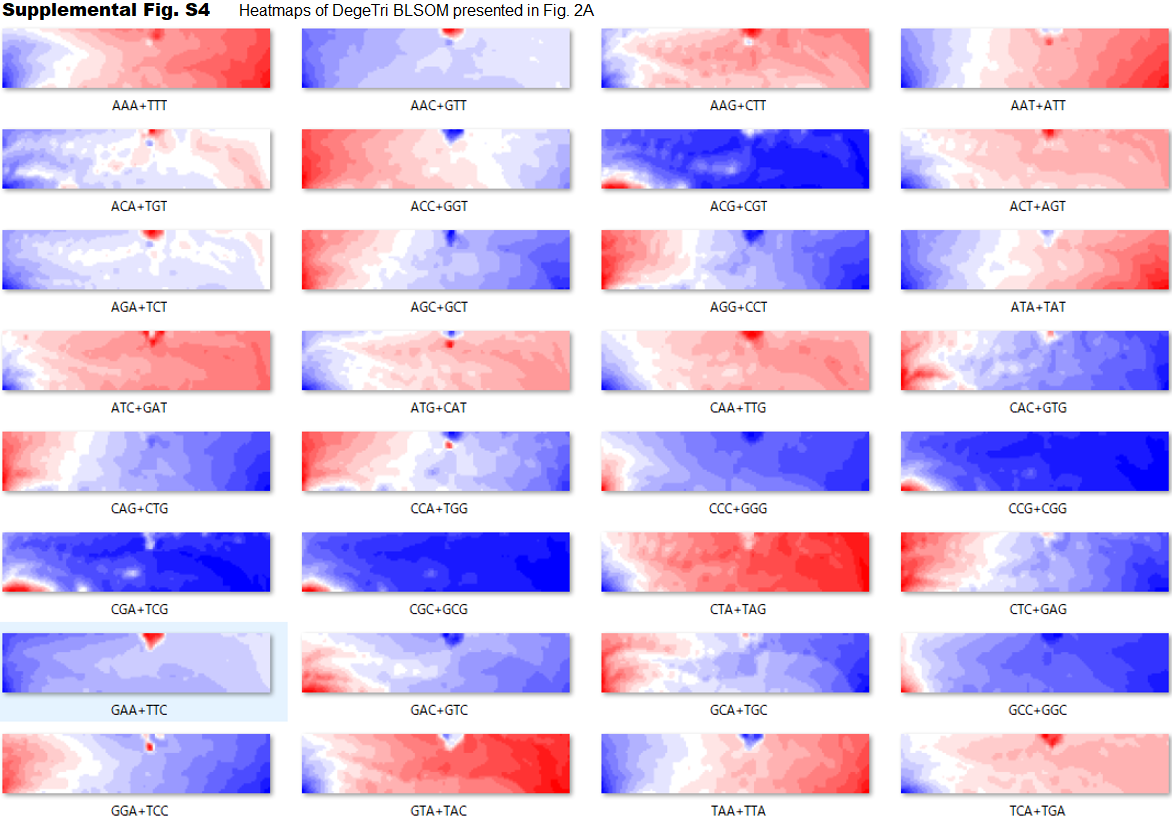


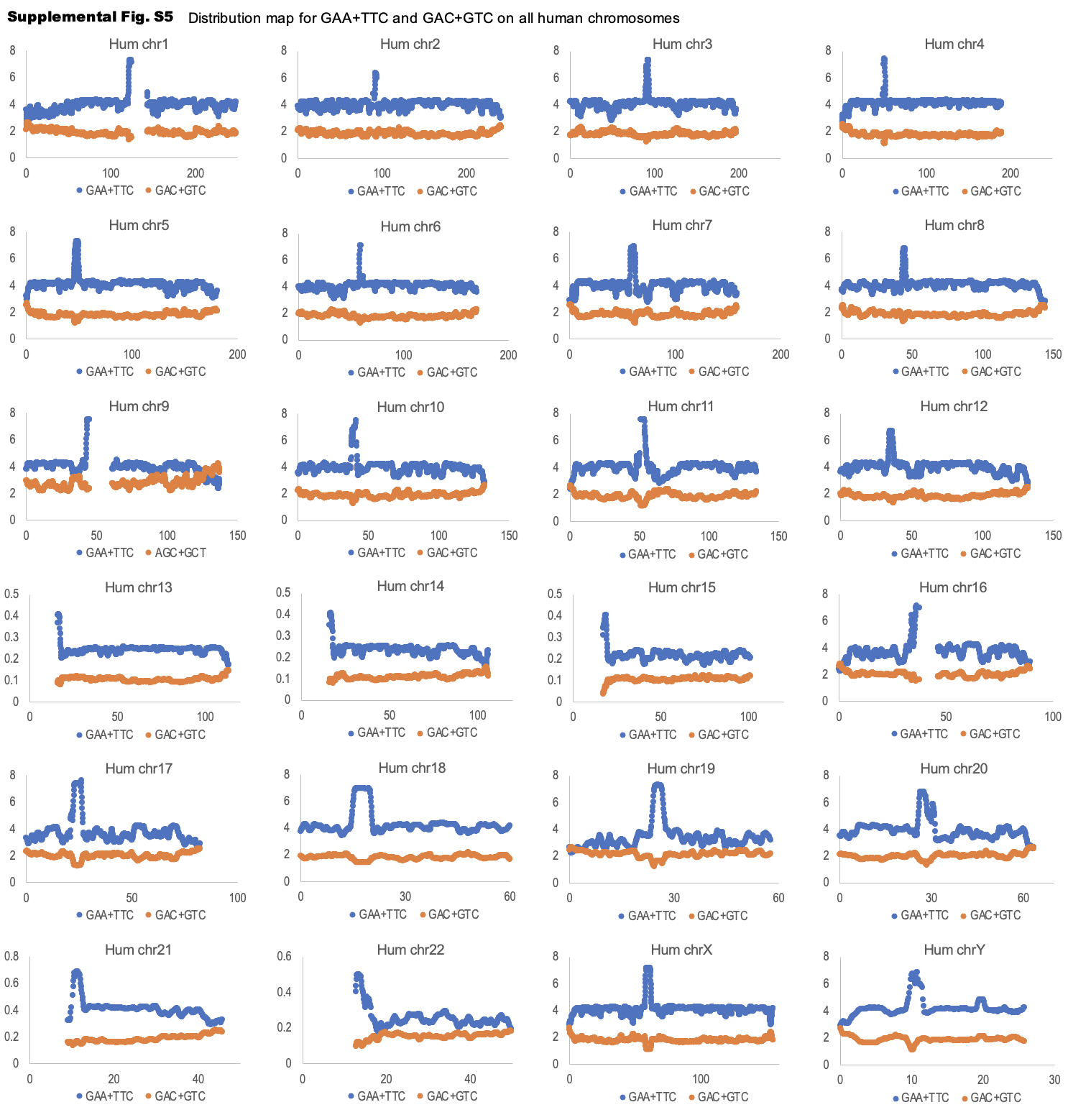


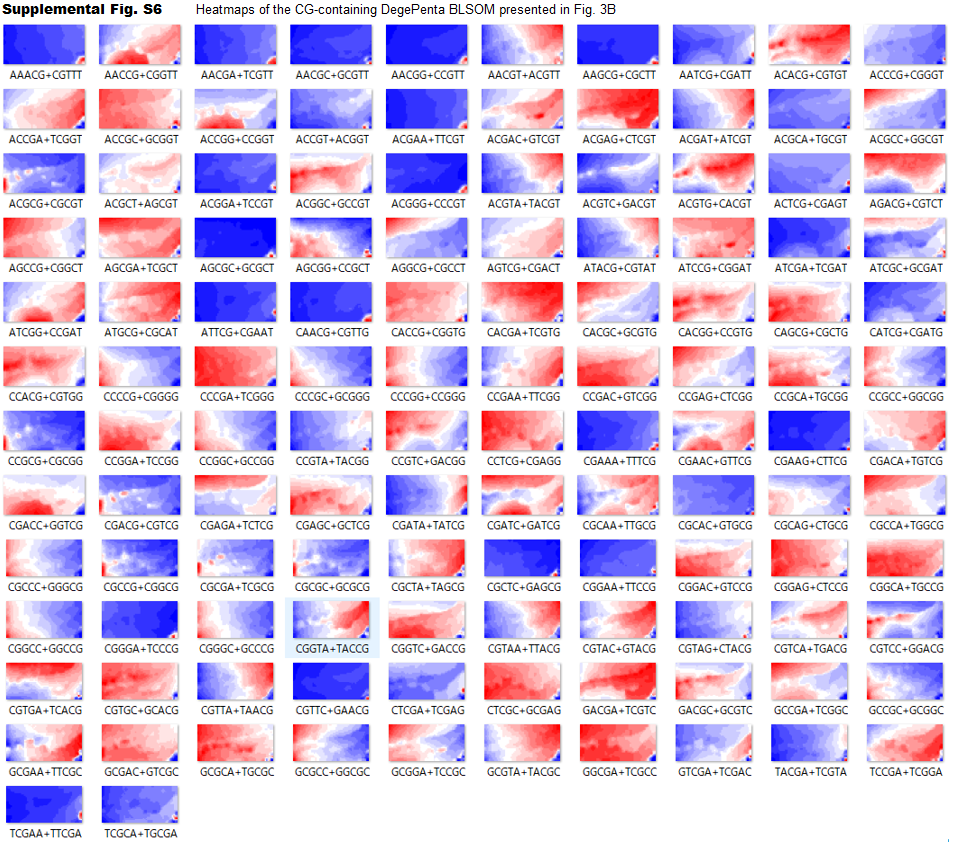


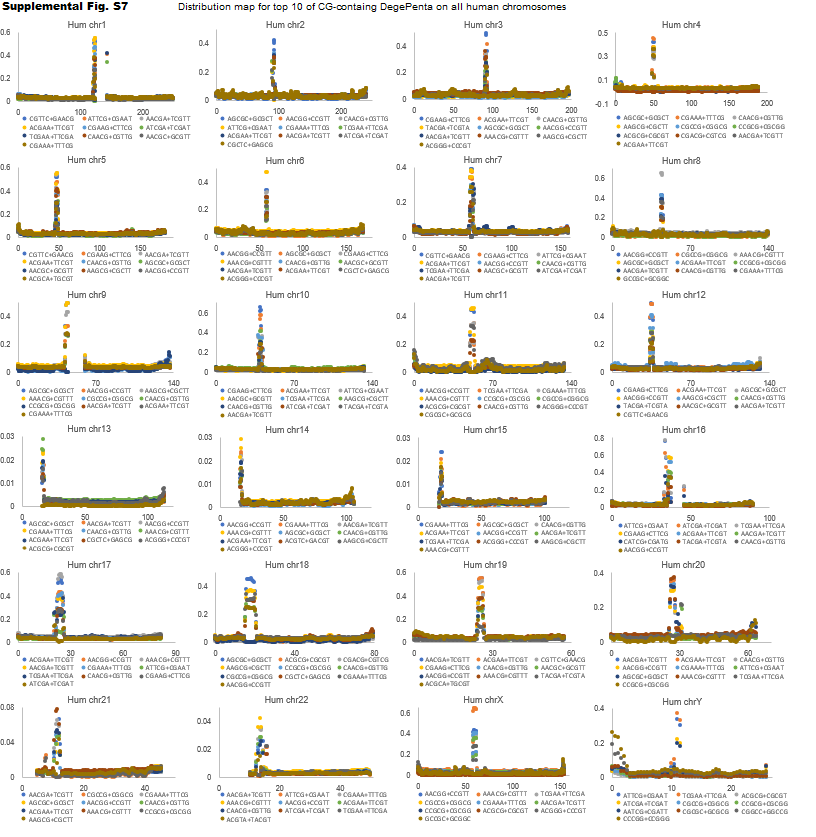


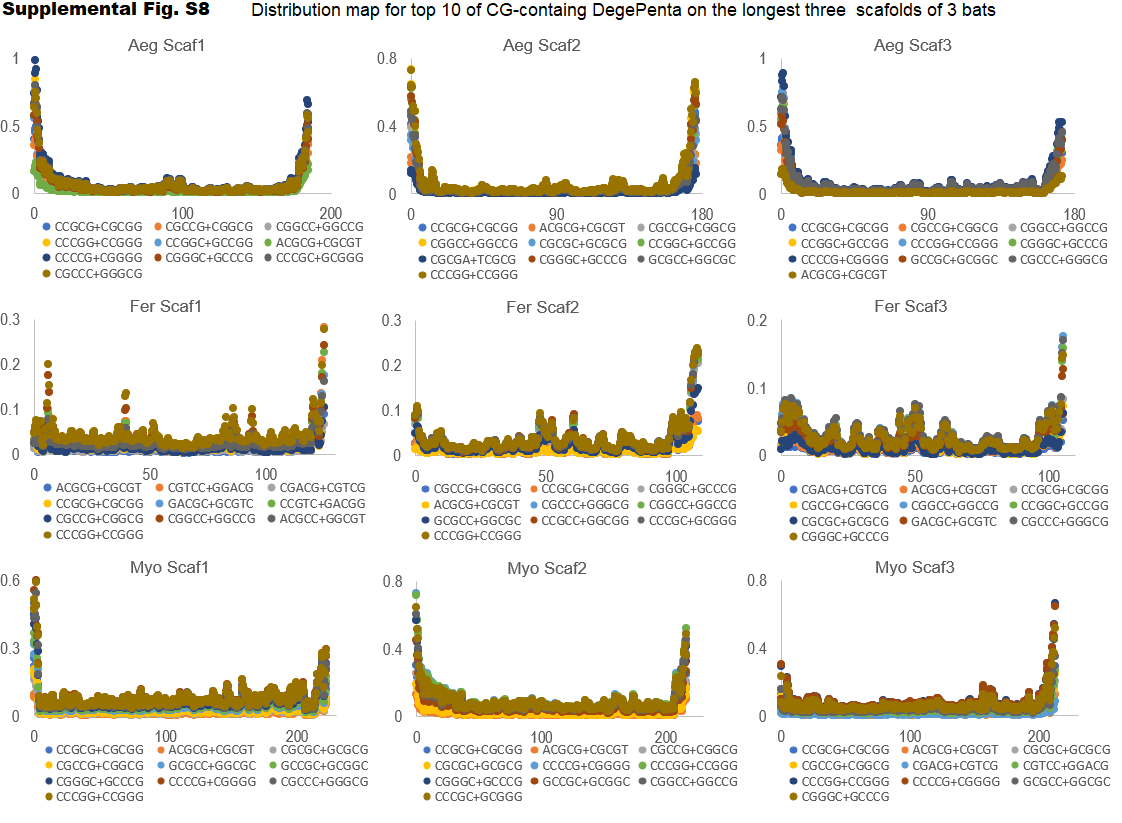


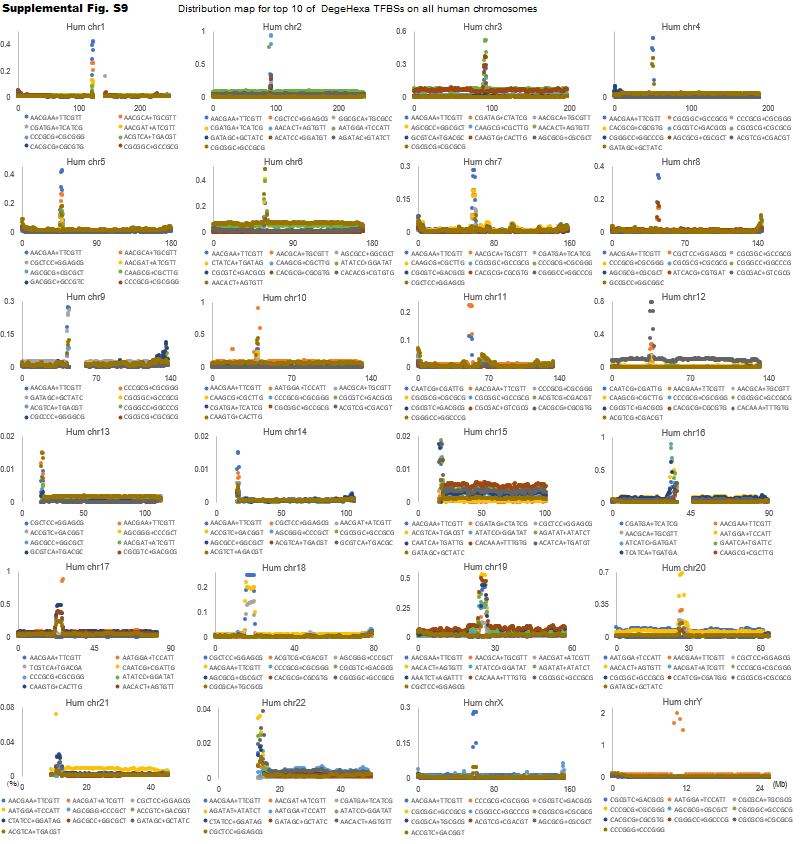


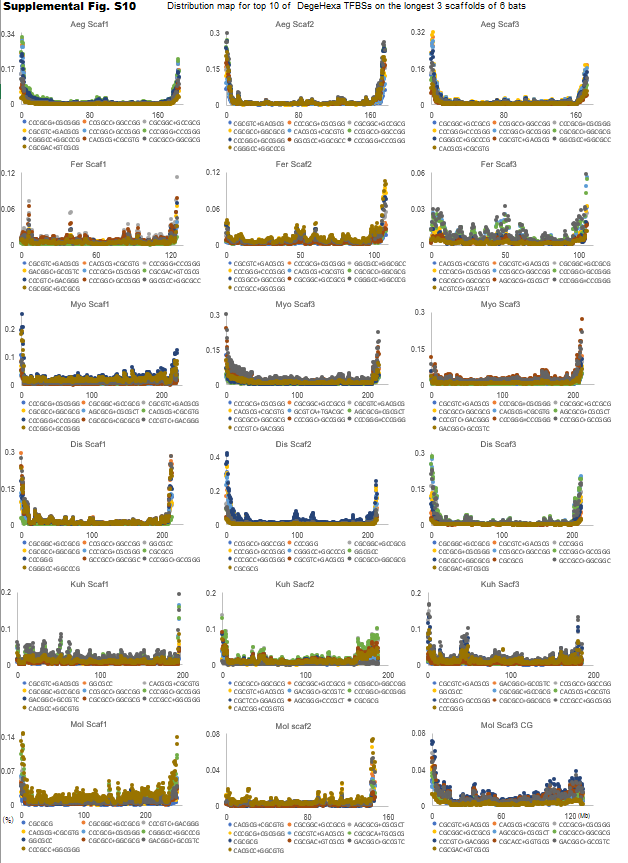


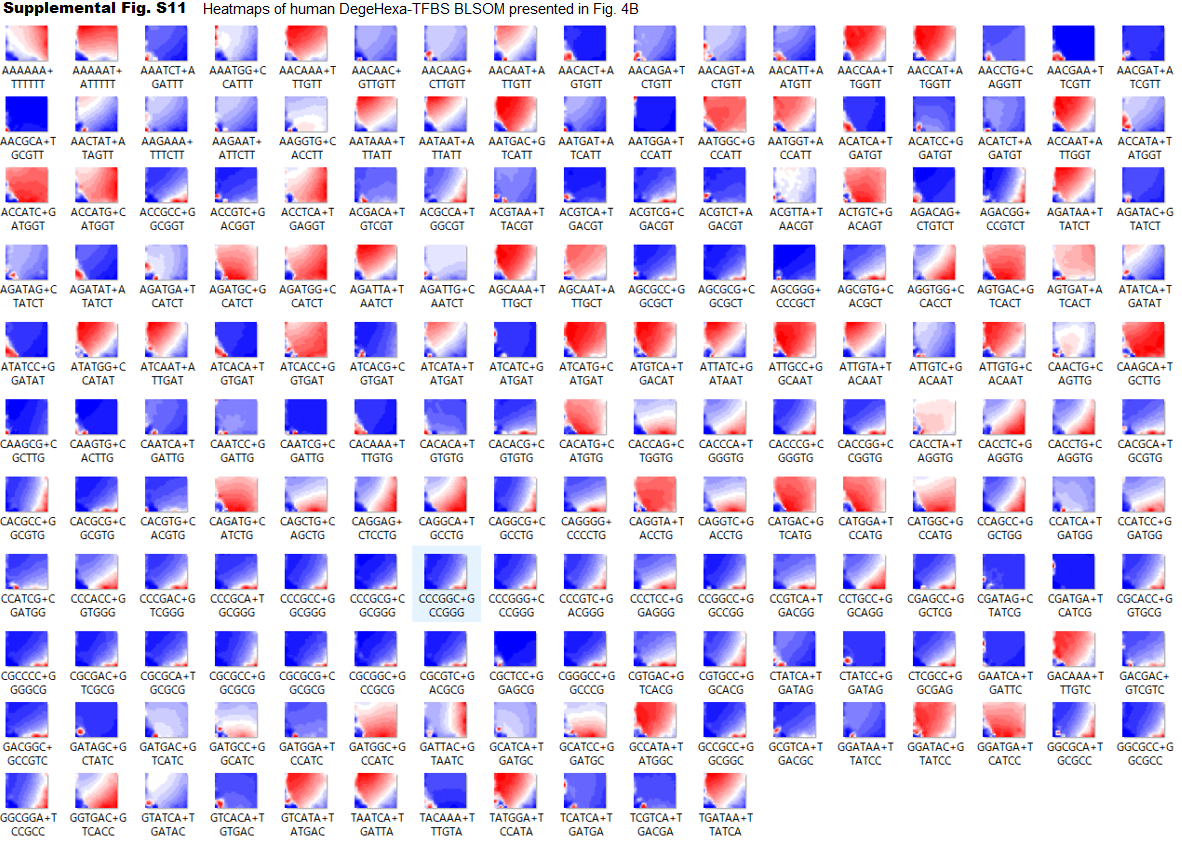

Supplement: Supplementary file 1 — Additional file 1: Supplemental Fig. S1. Distribution map for AC+GT and GA+TC on all human chromosomes.Supplemental Fig. S2. Distribution map for CG on longest 3 scaffolds of 6 bats.Supplemental Fig. S3. Distribution map for Odds for CG on longest 3 scaffolds of 3 bats.Supplemental Fig. S4. Heatmaps of DegeTri BLSOM presented in Fig. 2A.Supplemental Fig. S5. Distribution map for GAA+TTC and GAC+GTC on all human chromosomes.Supplemental Fig. S6. Heatmaps of CG-containing DegePenta BLSOM presented in Fig. 3B.Supplemental Fig. S7. Distribution map for top 10 of CG-containing DegePenta on all human chromosomes.Supplemental Fig. S8. Distribution map for top 10 of CG-containing DegePenta on longest three scaffolds of 3 bats.Supplemental Fig. S9. Distribution map for top 10 of DegeHexa TFBSs on all human chromosomes.Supplemental Fig. S10. Distribution map for top 10 of DegeHexa TFBSs on longest 3 scaffolds of 6 bats.Supplemental Fig. S11. Heatmaps of human DegeHexa-TFBS BLSOM presented in Fig. 4B. [file 12864_2022_8664_MOESM1_ESM.docx]
