# Supplementary material for: Comparative genomic analysis of the human genome and six bat genomes using unsupervised machine learning: Mb-level CpG and TFBS islands
Source: BMC Genomics. 2022 Jul 8;23:497. doi: 10.1186/s12864-022-08664-9 (PMC9264310; doi:10.1186/s12864-022-08664-9)
Supplement: Supplementary file 2 — Additional file 2: Supplemental Table S1. Human DegeHexa TFBS sequences. Supplemental Table S2. List of Sz regions in each human chromosome. [file 12864_2022_8664_MOESM2_ESM.docx]

Supplemental Table S1. Human DegeHexa TFBS sequences.

CCCGCC+GGCGGG, CACCTG+CAGGTG, CGCGCC+GGCGCG, GATGGC+GCCATC, CATGGC+GCCATG, AGATGG+CCATCT, CACGCG+CGCGTG, AACAAA+TTTGTT, CCATCC+GGATGG, ACGTCA+TGACGT, AATGGC+GCCATT, CCATCA+TGATGG, CATGAC+GTCATG, AATGAC+GTCATT, ACCATC+GATGGT, GATGAC+GTCATC, AACAAT+ATTGTT, CACGCA+TGCGTG, CGCGCG+CGCGCG, GCGTCA+TGACGC, CACGCC+GGCGTG, ATCATC+GATGAT, GGCGCC+GGCGCC, GATGGA+TCCATC, CCCGCG+CGCGGG, ATTGTC+GACAAT, AGATAG+CTATCT, ACATCA+TGATGT, CTATCA+TGATAG, GTCATA+TATGAC, CAGGTA+TACCTG, ACCATG+CATGGT, GCCATA+TATGGC, CTATCC+GGATAG, CGCGGC+GCCGCG, GGTGAC+GTCACC, AATGGT+ACCATT, GCCGCC+GGCGGC, ATCATG+CATGAT, AGATGA+TCATCT, CCATCG+CGATGG, AATGAT+ATCATT, AGCGTG+CACGCT, GACAAA+TTTGTC, CAGATG+CATCTG, CCCGGC+GCCGGG, CAAGTG+CACTTG, CACCTC+GAGGTG, AATAAT+ATTATT, CGTGAC+GTCACG, CAGCTG+CAGCTG, AGATAA+TTATCT, AGTGAC+GTCACT, ATTGTG+CACAAT, CAGGCG+CGCCTG, GGATGA+TCATCC, TCATCA+TGATGA, CACACA+TGTGTG, CACGTG+CACGTG, AAGGTG+CACCTT, AGCGCC+GGCGCT, CAGGAG+CTCCTG, CAGGTC+GACCTG, AACCTG+CAGGTT, CACCCG+CGGGTG, CACCAG+CTGGTG, CACCTA+TAGGTG, CAGGGG+CCCCTG, AGATTG+CAATCT, ATCATA+TATGAT, CACACG+CGTGTG, AGCAAT+ATTGCT, AATAAA+TTTATT, GACGGC+GCCGTC, TGATAA+TTATCA, CAATCA+TGATTG, CACCGG+CCGGTG, AACAGT+ACTGTT, AATGGA+TCCATT, ACCATA+TATGGT, GGATAA+TTATCC, CACATG+CATGTG, GCATCA+TGATGC, CATGGA+TCCATG, CAACTG+CAGTTG, ACCGCC+GGCGGT, CAATCC+GGATTG, AACAAC+GTTGTT, AGATGC+GCATCT, CACAAA+TTTGTG, CGATAG+CTATCG, GTCACA+TGTGAC, AACAAG+CTTGTT, GGCGCA+TGCGCC, CTCGCC+GGCGAG, ACATCT+AGATGT, AGCAAA+TTTGCT, AGCGCG+CGCGCT, GATAGC+GCTATC, ATCACC+GGTGAT, GGCGGA+TCCGCC, CGTGCC+GGCACG, CCCTCC+GGAGGG, CGCGTC+GACGCG, CAAGCA+TGCTTG, GCATCC+GGATGC, AGATAC+GTATCT, ATCACG+CGTGAT, CCCACC+GGTGGG, ATGTCA+TGACAT, CGCACC+GGTGCG, CCGGCC+GGCCGG, CGCTCC+GGAGCG, CGAGCC+GGCTCG, AACAGA+TCTGTT, AGTGAT+ATCACT, CAAGCG+CGCTTG, CGGGCC+GGCCCG, AACGAT+ATCGTT, ACATCC+GGATGT, AGATAT+ATATCT, CCAGCC+GGCTGG, CGATGA+TCATCG, CGCCCC+GGGGCG, AACATT+AATGTT, AGATTA+TAATCT, CGCGCA+TGCGCG, ACGTAA+TTACGT, CCTGCC+GGCAGG, AAAAAT+ATTTTT, ACCGTC+GACGGT, ACGTCG+CGACGT, AGCGGG+CCCGCT, CCCGTC+GACGGG, GTATCA+TGATAC, TATGGA+TCCATA, ACCAAT+ATTGGT, ATCACA+TGTGAT, ATTATC+GATAAT, ATTGTA+TACAAT, CGCGAC+GTCGCG, GGATAC+GTATCC, TCGTCA+TGACGA, AACCAA+TTGGTT, AAGAAT+ATTCTT, ACGCCA+TGGCGT, AGACAG+CTGTCT, ATATCC+GGATAT, ATTGCC+GGCAAT, CAATCG+CGATTG, CCCGGG+CCCGGG, AAATGG+CCATTT, AACACT+AGTGTT, AACCAT+ATGGTT, AACTAT+ATAGTT, AAGAAA+TTTCTT, ACCTCA+TGAGGT, ACGACA+TGTCGT, ATATCA+TGATAT, CACCCA+TGGGTG, CAGGCA+TGCCTG, CCCGAC+GTCGGG, CCCGCA+TGCGGG, CCGTCA+TGACGG, GATTAC+GTAATC, TAATCA+TGATTA, AAAAAA+TTTTTT, AAATCT+AGATTT, AACGAA+TTCGTT, AACGCA+TGCGTT, ACGTCT+AGACGT, ACGTTA+TAACGT, ACTGTC+GACAGT, AGACGG+CCGTCT, AGGTGG+CCACCT, ATATGG+CCATAT, ATCAAT+ATTGAT, GAATCA+TGATTC, GACGAC+GTCGTC, GATGCC+GGCATC, TACAAA+TTTGTA

**Supplemental Table S2** List of Sz regions in each human chromosome**
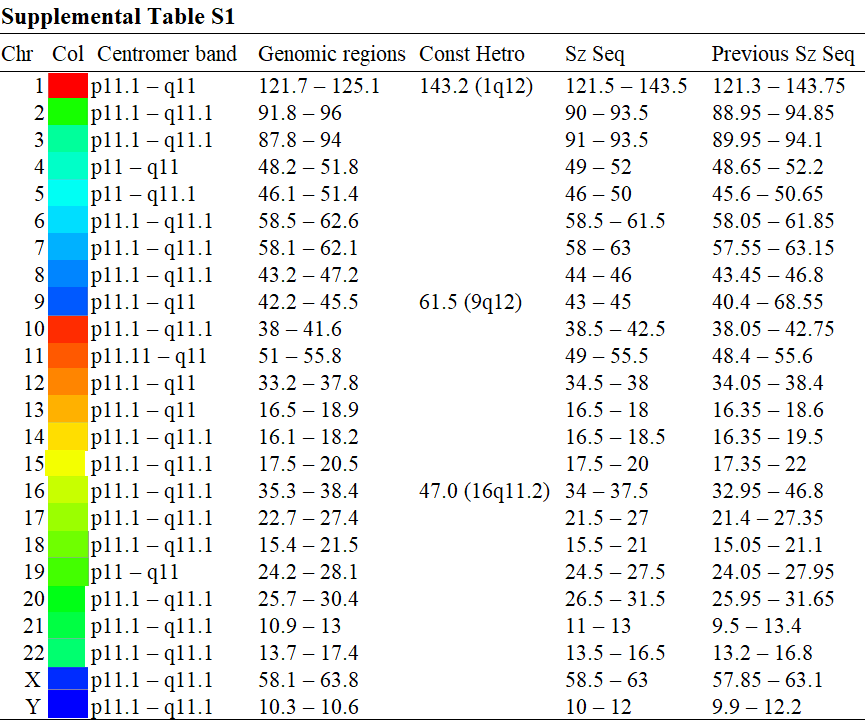
**

**Legend to Supplemental Table S2.** Colors used for each human chromosome and genomic regions of centromeric and pericentromeric constitutive heterochromatins and of Sz sequences. Abbreviations in the first column are as follows: Chr, chromosome: Col, Colors used for each human chromosome in Figs. 3C and 4B: Centromere band, centromere bands for p and q arms (https://genome.ucsc.edu): Genomic regions, regions of the centromere bands (Mb), whose central position is indicated by a magenta vertical bar in Figs. 1E, 2C, 3E, and 4D: Const Hetro, End position of a large-scale constitutive heterochromatin adjacent to the centromere band (https://genome.ucsc.edu): Sz Seq, genomic regions of Sz sequences for the TFBS DegeHexa-BLSOM in Fig. 4B: Previous Sz Seq, genomic regions of Sz sequences for the TFBS DegeOcta-BLSOM (1-Mb window sliding with a 50-kb step) previously published (Wada et al., 2020a). Notably, the main difference between the present and previous Sz results was found mainly in the large heterochromatin regions of chr9 and 16 (9q12 and 16q11.2). This difference may be due to the use of 181 DegeHaxa TFBSs in the present study and 3946 DegeOcta TFBSs in the previous study and relate to the chromosome-dependent TFBS enrichment in the constitutive heterochromatin regions.
